# Not Breathing During the Approach Phase Ameliorates Freestyle Turn Performance in Prepubertal Swimmers

**DOI:** 10.3389/fspor.2021.731953

**Published:** 2021-10-05

**Authors:** Emanuela Faelli, Laura Strassera, Sara Ottobrini, Vittoria Ferrando, Ambra Bisio, Luca Puce, Marco Panascì, Cesare Lagorio, Piero Ruggeri, Marco Bove

**Affiliations:** ^1^Department of Experimental Medicine, Section of Human Physiology, University of Genoa, Genoa, Italy; ^2^Centro Polifunzionale di Scienze Motorie, University of Genoa, Genoa, Italy; ^3^Laboratory of Adapted Motor Activity (LAMA), Department of Public Health, Experimental Medicine and Forensic Science, University of Pavia, Pavia, Italy; ^4^Laboratory for Rehabilitation Medicine and Sport (LARMS), Rome, Italy; ^5^Department of Neuroscience, Rehabilitation, Ophthalmology, Genetics, Maternal and Child Health, University of Genoa, Genoa, Italy; ^6^IRCCS Ospedale Policlinico San Martino, Genoa, Italy

**Keywords:** swimming performance, freestyle turn, kinematic parameters, video analysis, prepubertal swimmers

## Abstract

This study compared the effects of two breathing conditions during the freestyle turn approach phase in swimmers. Thirty-four prepubertal swimmers (mean ± SD: 10.59 ± 0.97 years) were divided into two groups: No Breath (NB), not breathing at the last stroke, and Breath Stroke (BS). Swimmers performed three turns with 5 min of rest between the repetitions. Kinematic parameters were recorded with two underwater and two surface cameras. Total turn time (NB: 9.31 ± 1.34 s; BS: 10.31 ± 1.80 s; *p* = 0.049), swim-in time (NB: 3.89 ± 0.63 s; BS 4.50 ± 0.79 s; *p* = 0.02) and rotation time (NB: 2.42 ± 0.29 s; BS: 3.03 ± 0.41 s; *p* = 0.0001) were significantly shorter and swim-in distance [NB: 0.70 (0.58,0.77) m; BS: 0.47 (0.34,0.55) m; *p* = 0.0001], glide distance (NB: 1.06 ± 0.21 m; BS: 0.70 ± 0.20 m; *p* = 0.0001) and surfacing distance [NB: 1.79 (1.19,2.24) m; BS: 1.18 (0.82,1.79) m; *p* = 0.043] were significantly longer in NB than in BS. Moreover, speed-in (NB: 1.04 ± 0.14 m/s; BS: 0.93 ± 0.14 m/s; *p* = 0.031) and push-off speed (NB: 2.52 ± 0.30 m/s; BS: 1.23 ± 0.20 m/s; *p* = 0.001) were significantly higher in NB than in BS. Swim-in time was positively and negatively correlated with rotation time and glide distance, respectively, whilst negative relationships between total turn time and swim-in distance, total turn time and surfacing distance and total turn time and speed-in were found. Our study showed that in prepubertal swimmers not breathing at the last stroke during the approach phase positively affected kinematic parameters of the turn, allowing to approach the wall faster, rotate the body quicker, increase push-off speed, reduce turn execution time, thus improving overall turn performance.

## Introduction

The swim race consists of start, clean swimming (or swim stroke), turns and finish. Previous studies showed that in freestyle races swimmers spend from ≈20 to ≈37% of total race time in executing swimming turns in 100 and 1,500 m races, respectively (Morais et al., 2019, 2020). The high relevance of turn outcome in swimming performances suggests that coaches and swimmers should dedicate a significant portion of the training to perfect this action.

During the turn, swimmers must reverse the direction of the body in the shortest time and regain the speed in the opposite direction (Blanksby et al., 1996). The tumble turn, also known as freestyle turn, involves different phases: the approach to the wall, the turn or rotation to reorient the body in preparation for swimming the next lap (tumble), the push-off or wall-contact, the glide, the underwater propulsion and the stroke resumption (Puel et al., 2012; Weimar et al., 2019).

A successful turn performance depends on a number of kinematic parameters within these different phases. Considering that the turn outcome significantly contributes to overall swimming performance (Morais et al., 2019, 2020), it is of great importance to identify what variables can enhance turning skill.

Scientific literature reported a number of studies examining the different phases of the turn and their most characterizing parameters, such as rotation time during the tumble (Rejman and Borowska, 2008), peak force and wall contact time during the push-off (Araujo et al., 2010; Nicol et al., 2019; Weimar et al., 2019) and velocity and displacement covered during the gliding phase (Zamparo et al., 2012; Marinho et al., 2020). However, to the best of our knowledge, most of these studies involved elite swimmers, whereas only limited information is available on turn performance in young swimmers. A previous study demonstrated that in young swimmers a greater head-wall distance at rotation was associated with fastest turns, showing a negative relationship during the approach phase between this parameter (defined the swim-in distance) and total turn time (Blanksby et al., 1996). The approach to the wall is the first phase of the freestyle turn and, during this phase, swimmers must proceed towards the wall at high speed, in order to have a strong push in the next wall-contact phase. More recently, Puel et al. (2012) confirmed, in elite swimmers, the importance of a longer head-wall distance at rotation during the approach to the wall.

In the early stages of training, coaches usually start the teaching the freestyle turn, learning the different phases separately and offering children more strategies and exercises to increase their motor skill level (Federazione Italiana Nuoto, 2014). For example, while learning the approach to the wall, coaches usually show prepubertal swimmers different breathing exercises to identify the most effective breathing technique to adopt before turning (Federazione Italiana Nuoto, 2014). Nevertheless, no studies investigated in young swimmers whether different breathing conditions significantly affect turn performance. This aspect could be particularly relevant during the developmental years, in which young swimmers must build and consolidate a specific and detailed motor pattern of the turn.

Hence, to this aim, we examined in prepubertal swimmers with a similar swimming experience the effects induced by two different breathing techniques (not breathing at the last stroke vs. breathing at the last stroke) on selected kinematic features of freestyle turn phases and on turn performance. We hypothesized that not breathing at the last stroke during the approach to the wall could positively influence the turning performance, that in turn represents an important component in overall swimming performance.

## Materials and Methods

### Participants

Thirty-four prepubertal swimmers (17 males and 17 females), with at least 6 h/week of training volume and 5 years of swimming experience, were recruited. Participants were divided into two groups, on the basis of the preferred breathing technique at the last stroke before turning: No Breath (NB) and Breath Stroke (BS). In the NB group (*n* = 17), prepubertal swimmers did not breathe at the last stroke during the approach phase, while in the BS group (*n* = 17) participants breathed. Two out 17 of participants of the NB group did not complete the experimental protocol. No significant differences between groups concerning age, gender, years of swimming practice, anthropometric measures and 50 m swim time were found (Table 1).

**Table 1:** Participants' characteristics of No Breath (NB) and Breath Stroke (BS) groups.

	**NB group (*n* = 17)**	**BS group (*n* = 17)**
Age (years) (min, max)	10.60 ± 1.06 (9, 13)	10.59 ± 0.94 (9, 12)
Sex (M, F)	8 M, 9 F	9 M, 8 F
Swimming Experience (years)	5.67 ± 1.59	5.59 ± 2.03
Hours/ Week (h/wk)	8.40 ± 2.92	7.56 ± 2.65
Height (m)	1.43 ± 0.07	1.43 ± 0.08
Body Mass (kg)	34.17 ± 4.41	35.41 ± 5.69
BMI (kg/m^2^)	16.78 ± 1.65	17.15 ± 1.64
Right arm (m)	0.48 ± 0.03	0.48 ± 0.04
Right arm+hand (m)	0.64 ± 0.04	0.64 ± 0.05
Left arm (m)	0.48 ± 0.03	0.48 ± 0.04
Left arm+hand (m)	0.64 ± 0.04	0.63 ± 0.05
Right leg (m)	0.81 ± 0.06	0.81 ± 0.06
Left leg (m)	0.81 ± 0.06	0.81 ± 0.06
Right foot (m)	0.22 ± 0.01	0.22 ± 0.02
Left foot (m)	0.22 ± 0.02	0.22 ± 0.03
50 m time (s)	41.5 (37.5, 47.6)	42.97 (39.4, 48.9)

*Data are reported as mean ± SD values in case of normal distribution or median (interquartile interval) values in case of not-normal distribution*.

Before entering the study, prepubertal swimmers' parents were fully informed about the study aims and procedures. Participants and their legal guardians provided written informed consent. The experimental protocol was conformed to the code of Ethics of the World Medical Association (Declaration of Helsinki). The local ethics committee of the University of Genoa approved the study (Comitato Etico per la Ricerca di Ateneo, Genoa, Italy, No. 2020/21).

### Sample Size

Estimation of sample size was performed using the GPower software (3.1 software Düsseldorf, Germany) applying an a-priori two-sided power analysis. This calculation generated a desired sample size of at least 15 subjects for each group. However, we recruited 34 participants, 17 in the NB group and 17 in the BS group, to allow for drop-out during the intervention period (Faul et al., 2007).

### Experimental Design

Before the experimental protocol, tape markers, allowing the tracking of relocation of different segments of the body, were applied to the participants. Markers were located on both sides of the body on the head, shoulders, elbows, wrists, hips, knees and ankles of each swimmer. The experimental protocol was performed in a 25 m pool. Prior to the testing trials, swimmers warmed up with a 600 m swim including preparatory exercises for the experimental test, then they performed three freestyle turns as fast as possible, with 5 min of rest between the repetitions.

### Video Analysis Setting

**Figure 1:**
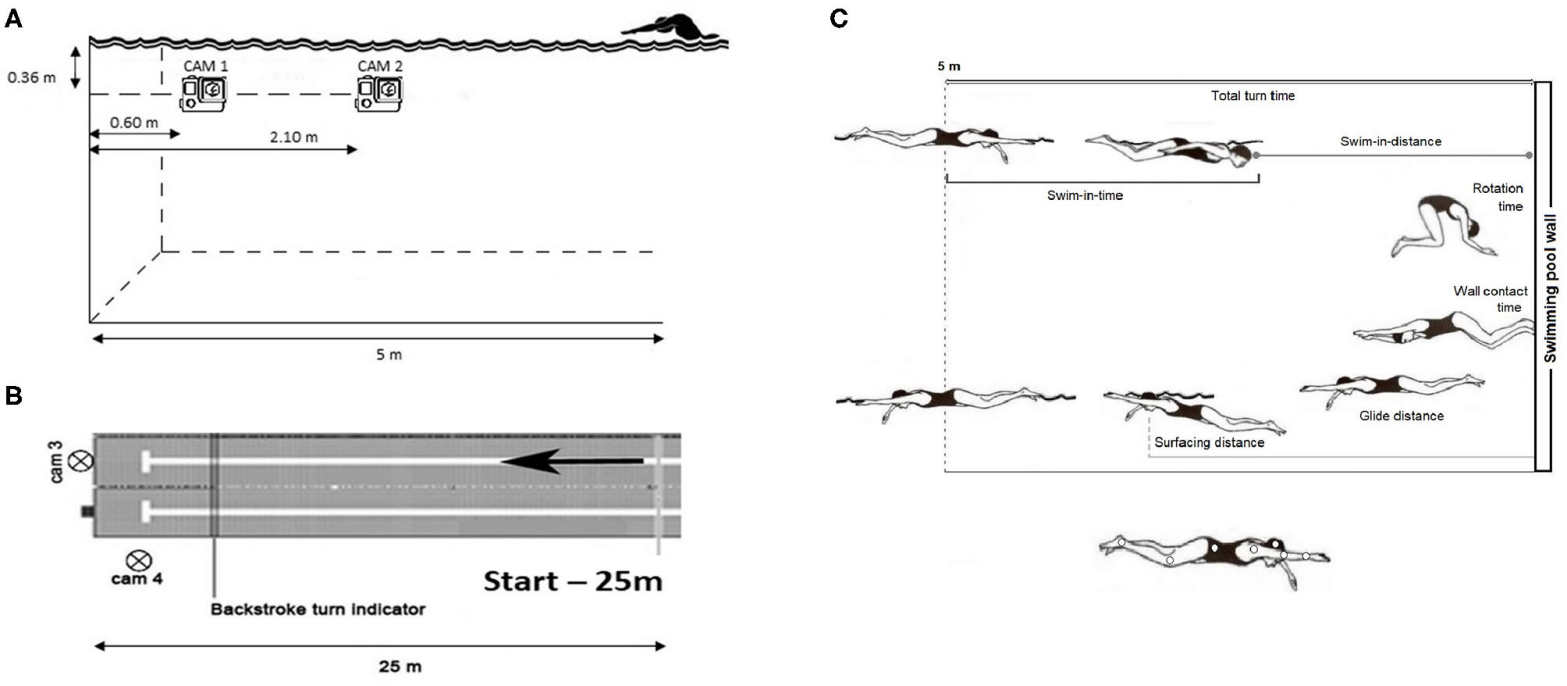
Position of the two underwater cameras **(A)** and of the two surface cameras **(B)**; outcome variables and markers position **(C)** for video analysis.

A 2D video analysis was performed using Kinovea software 0.8.15 (Copyright © 2006–2011, Joan Charmant & Contrib). Each trial was recorded by four cameras (two underwater and two surface-fixed) (GoPro® HERO5, 60Hz) at 120 fps and with a resolution of 720 pixel. The two underwater cameras were positioned with the suction cup on a Plexiglas panel fixed to the lateral wall of the swimming pool. Both cameras were located at a depth of 0.36 m, at a distance of 0.6 m and 2.10 m from the turning wall, respectively (Figure 1A).

In order to obtain a frontal view of the swimmer, a third surface camera was placed on the board of the swimming pool, to a height of 0.30 m from the edge and with a downward inclination of 45°. The fourth surface camera was positioned above the lateral wall of the pool, on a ladder situated at a distance of 1.31 m from the turning wall and at a height of 1.87 m from the floor (Figure 1B). A distance of 5 m from the swimming pool wall was assumed as the turn distance (Blanksby et al., 1996; Rejman and Borowska, 2008). Moreover, a black rubber band on the rope in the pool lane, 5 m away from the turning wall, was fixed in water, as a reference point for the video analysis of the selected kinematic variables.

### Outcome Measures

The video analysis was carried out by a researcher blinded to the aim of the study.

Temporal, distance and speed parameters of the freestyle turn phases were chosen for the performance analysis (Figure 1C). Parameters' specification and description (Rejman and Borowska, 2008; Puel et al., 2012) are reported in Table 2. Data used in the statistical analysis correspond to the average data over the three turn repetitions and to their coefficient of variability (CV).

**Table 2:** Parameters' specification and description and reference markers.

**Parameters**		**Definitions**
**Time**	Total turn time (s)	Time period from the moment when the hip joints pass through the point placed 5 m from the wall before turning, till the moment when the hip joints pass through the point placed 5 m from the wall after turning. Reference marker: hip.
	Swim-in time (s)	Time period from the moment when the hip joints pass through the point placed 5 m from the wall before turning, till the moment of the turning initiation (downward movement of the head). Reference markers: hip, head.
	Rotation time (s)	Time period from the moment of the turning initiation, till the moment when the turning is finished (first moment of the feet contact with the wall). Reference markers: wrist and ankle.
	Wall contact time (s)	Time period from the first feet contact with the wall, till the moment when the feet lost contact with the wall. Reference marker: ankle.
**Distance**	Swim-in distance (m)	Head to wall distance at the start of the rotation. Reference marker: head.
	Glide distance (m)	Distance of the hip joints displacement between the moment when the feet lost contact with the wall and the moment of the first propulsive movement initiation. Reference marker: hip.
	Surfacing distance (m)	Distance of the hip joints displacement between the moment when feet lost contact with the wall, and the moment of the surfacing. Reference marker: hip.
**Speed**	Speed-in (m·s^−1^)	Average speed from when the hip is 5 m from the wall to the first contact of the feet to the wall. Reference marker: hip.
	Speed-out (m·s^−1^)	Average speed since the last contact of the feet to the wall up to 5 m. Reference marker: hip.
	Push-off speed (m·s^−1^)	Speed at the end of push-off calculated at hip joints. Reference marker: hip.
**Angle**	Push-off angle (°)	Angle described by the markers positioned on the head, hip and ankle at the instant of push-off.

### Statistical Analysis

The distribution of the outcome parameters was tested by means of Shapiro-Wilk test. Total turn time (s), swim-in time (s), rotation time (s), wall-contact time (s), glide distance (m), speed-in (m·s^−1^), speed-out (m·s^−1^), push-off speed (m·s^−1^) and push-off angle (°) were normally distributed, whilst swim-in distance (m) and surfacing distance (m) were not normally distributed. All CV values were not normally distributed.

The comparison between NB and BS groups was performed by means of independent *t*-tests in case of normally-distributed data, and Mann-Whitney test in case of not normally-distributed data. The analyses were performed using IBM SPSS STATISTIC, version 20 for Windows. The level of significance was set at *p* = 0.05. In this study, kinematic parameters are reported as mean value ± standard error associated with Hedges's index (*g*)—a measure of effect size (Tomczak and Tomczak, 2014), in case of normal distribution, and median value (interquartile interval) associated with the eta squareη^2^–a measure of effect size (Tomczak and Tomczak, 2014), when not normally distributed. CV values, computed for each variable as standard deviation/mean^*^100, are reported as means values and 95% CI.

Pearson's correlations were applied to evaluate the relationship between swim-in time and rotation time, swim-in time and glide distance, and speed-in and total turn time. Spearman's correlation was used to check for relationships between the total turn time and the swim-in distance, and the total turn time and the surfacing distance. Correlations were evaluated considering data from both groups pooled together, and considering data from the two groups separately. Bonferroni's correction for multiple comparisons was applied. For this reason, the significance level was set at *p* = 0.05/2 =0.025.

## Results

### Kinematic Parameters

The statistical analyses showed that total turn time was significantly lower in NB group (9.31 ± 1.34 s) than in BS group (10.31 ± 1.80 s) [*t*_(30)_ = 29.89, *p* = 0.049, *g* = 0.58], as well as the swim-in time [NB group 3.89 ± 0.63 s; BS group 4.50 ± 0.79 s; *t*_(30)_ = −2.41, *p* = 0.02, *g* = 0.85], whereas the swim-in distance was significantly higher in the NB group [0.70 (0.58, 0.77) m] than in the BS group [(0.47 (0.34, 0.55) m] (*Z* = −3.69, *p* = 0.0001,η^2^ = 0.424). Rotation time was found to be significantly lower in NB (2.42 ± 0.29 s) than in BS (3.03 ± 0.41 s) group [*t*_(30)_ = −4.76, *p* = 0.0001, *g* = 1.69]. No difference appeared between the two groups in wall-contact time [NB group 0.57 ± 0.26 s; BS group 0.70 ± 0.25 s; *t*_(30)_ = −1.38, *p* = 0.18, *g* = 0.49]. Glide distance was significantly higher in NB group (1.06 ± 0.21 m) than in BS group (0.70 ± 0.20 m) [*t*_(30)_ = 4.06, *p* = 0.0001, *g* = 1.44] as well as the surfacing distance [NB group 1.79 (1.19, 2.24) m; BS group 1.18 (0.82, 1.79); *Z* = −2.02, *p* = 0.043,η^2^ = 0.128]. Speed-in was significantly higher in NB (1.04 ± 0.14 m/s) than in BS group (0.93 ± 0.14 m/s) [*t*_(30)_ = 2.26, *p* = 0.031, *g* = 0.80], whilst no significant difference was found in speed-out [NB group 1.30 ± 0.19 m/s; BS group 1.23 ± 0.20 m/s; *t*_(30)_ = 1.07, *p* = 0.3, *g* = 0.38]. Finally, push-off speed was significantly higher in NB (2.52 ± 0.30 m/s) than in BS (2.14 ± 0.30 m/s) group [*t*_(30)_ = 3.53, *p* = 0.001, *g* = 1.25], whilst no significant difference was present between groups in push-off angles, although the values of NB groups were closer to 180° than those of BS group [NB group 176 ± 7.27°; BS group 170 ± 12.56°; *t*_(30)_ = 1.51, *p* = 0.13, *g* = 0.62].

The analyses on CV values of all the previously mentioned parameters did not find any significant differences among groups. CV values are reported afterwards. CV Total turn time: NB 1.67 (1.21, 2.12) and BS 1.35 (0.87, 1.83) (*Z* = −1.21, *p* = 0.23,η^2^ = 0.046); CV Swim-in time: NB 3.53 (2.49, 4.58) and BS 3.29 (2.37, 4.22) (*Z* = −0.31, *p* = 0.76,η^2^ = 0.003); CV Rotation time NB: 5.87 (4.29, 7.45) and BS 4.82 (3.20, 6.45) (*Z* = −1.33, *p* = 0.18,η^2^ = 0.056); CV Wall-contact time: NB 10.07 (6.83, 13.30) and BS 9.88 (6.17, 13.60) (*Z* = −0.53, *p* = 0.60,η^2^ = 0.009); CV Swim-in distance: NB 12.67 (9.00, 16.33) and BS 16.06 (11.11, 21.021) (*Z* = −0.78, *p* = 0.44,η^2^ = 0.019); CV Glide distance: NB 12.00 (8.94, 15.06) and BS 13.59 (8.64, 18.54) (*Z* = 0.00, *p* = 1,η^2^ = 0.00); CV Surfacing distance: NB 6.53 (4.59, 8.47) and BS 7.00 (4.29, 9.71) (*Z* = −0.11, *p* = 0.91,η^2^ = 0.00); CV Speed-in: NB 6.60 (5.28, 7.92) and BS 0.06 (0.04, 0.09) (*Z* = −0.48, *p* = 0.63,η^2^ = 0.007); CV Speed-out: NB 5.07 (3.82, 6.31) and BS 6.35 (4.96, 7.75) (*Z* = −0.28, *p* = 0.78,η^2^ = 0.003); CV Push-off speed: NB 4.13 (3.17, 5.09) and BS 5.29 (4.21, 6.38) (*Z* = −1.72, *p* = 0.09,η^2^ = 0.092).

### Correlation Analysis

**Figure 2:**
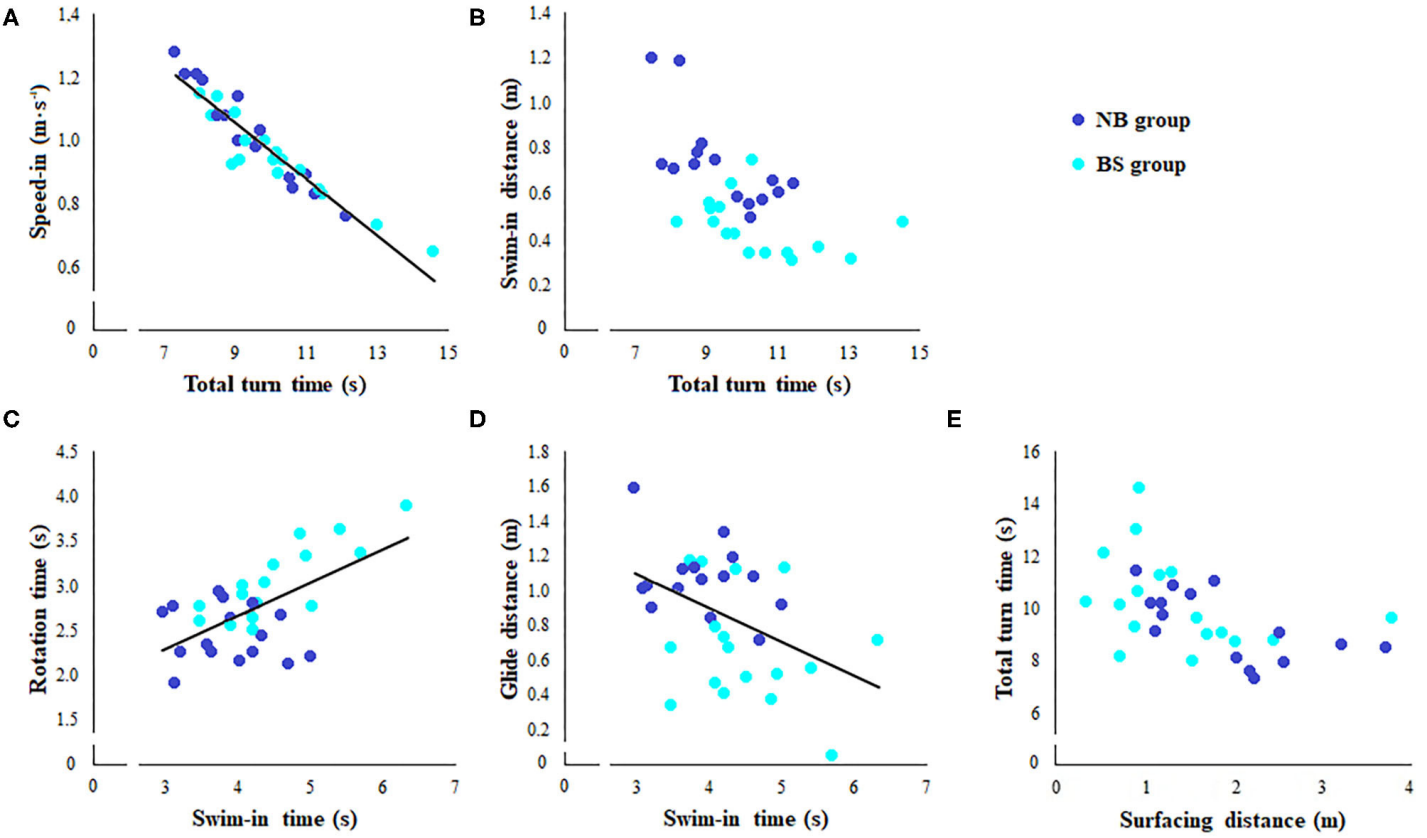
Relationships between kinematic parameters of the different turn phases for No Breath (NB—dark blue circles) and Breath Stroke (BS—light blue circles) groups' data. Each circle represents a single subject. When present, the line refers to linear regression analysis computed on the data of the two groups pooled together. **(A)** Speed-in vs. total turn time; **(B)** swim-in distance vs. total turn time; **(C)** rotation time vs. swim-in time; **(D)** glide distance vs. swim-in time; **(E)** surfacing distance vs. total turn time.

When considering the data from the two groups pooled together, significant negative relationships appeared between total turn time and surfacing distance (*R* = −0.66, *p* = 0.0003), and total turn time and swim-in distance (*R* = −0.59, *p* = 0.0006). Furthermore, a significant positive relationship was found between swim-in time and rotation time (*R* = 0.62, *p* = 0.0002). At last, significant negative relationships were observed between swim-in time and glide distance (*R* = −0.44, *p* = 0.01), and between speed-in and total turn time (*R* = −0.94, *p* = 0.0002) (Figure 2).

The correlation analysis performed separately on each group showed that the significant negative relationship between surfacing distance and total turn time was present in NB (*R* = −0.73, *p* = 0.002), whilst a trend towards the significance appeared in BS (*R* = −0.46, *p* = 0.07). The significant negative relationship between the total turn time and swim-in distance was observed for both groups (NB: *R* = −0.69, *p* = 0.004, BS: *R* = −0.60, *p* = 0.012). The significant positive relationship between swim-in time and rotation time was present only in BS group (*R* = 0.81, *p* = 0.0003). No significant relationship appeared between swim-in time and glide distance when considering the two groups separately. Finally, significant negative relationships were found in both groups between speed-in and total turn time (NB: *R* = −0.98, *p* = 0.0004, BS: *R* = −0.92, *p* = 0.0005).

## Discussion

The present study compared, for the first time, in prepubertal swimmers two breathing techniques (not breathing vs. breathing at the last stroke) during the freestyle turn approach phase, to investigate their possible effects both on kinematic parameters of the next turn phases and on overall turn performance.

According to a previous study (Blanksby et al., 1996), we adopted the total turn time over 5 m as turn performance test, since we considered that using the 50 m time could have masked some aspects of the turn technique, as 50 m swimming time includes some advantages from the first few meters at the start but also some negative effects related to fatigue over the final few meters (Blanksby et al., 1996).

In this study we demonstrated that in prepubertal swimmers not breathing at the last stroke during the approach phase induced positive effects on the kinematic parameters of the subsequent turn phases, thus improving the freestyle turn execution time. Freestyle turn involves a complex turning action that includes a main rotation around the transverse axis and on the sagittal plane, combined or not with a rotation around the other axis, especially the longitudinal one (Vilas-Boas and Fernandez, 2003), and it is typically divided into several phases. During the initial learning stages, young swimmers must achieve a motor competence for turn techniques (Federazione Italiana Nuoto, 2014), and errors within a single phase could affect kinematic parameters of the other turn phases, thus impairing turn performance (Hines, 2008).

### Effects on Kinematic Parameters of the Approach Phase

During the approach phase, the NB group showed a significantly higher speed-in value compared with the BS group, suggesting that not breathing at the last stroke allowed swimmers to maintain the head and the whole body in a hydrodynamic position without breaking the approach to the wall and therefore not losing speed during this phase.

In addition, the NB group showed a significantly higher swim-in distance with a significantly shorter rotation time and a swim-in time compared with the BS group, demonstrating that not breathing at the last stroke allows prepubertal swimmers to start the rotation farther away from the wall, thus reducing the turn time, as previously observed in young (Blanksby et al., 1996) and elite swimmers (Puel et al., 2012). This probably happened since all body segments turned simultaneously: the head did not move in advance with respect to the body, and feet, hips, shoulders and head were aligned during the contact of the feet with the wall, resulting in an advantageous position for the subsequent push-off phase. Indeed, the mean value of the push-off angle of the NB group was closer to 180° than that of the BS group, suggesting that head, hips and feet of swimmers not breathing at the last stroke were more aligned than the others. However, the analysis on the push-off angle did not reveal a significant difference between groups, but this aspect could be probably linked to the high variability of the BS group.

Moreover, the time needed to rotate the head and breathe would explain the higher distance covered by the athletes of the BS group and, therefore, their shorter swim-in distance.

### Effects on Kinematic Parameters of the Push-Off Phase

The push-off speed value of the NB group was significantly higher than in the BS group, showing that swimmers who did not breathe at the last stroke were able to maintain high speed values not only in the approach phase but also in the subsequent phases.

### Effects on Kinematic Parameters of the Underwater Phase

Our results suggest that not breathing at the last stroke during the approach phase can positively influence the kinematic variables of the underwater phase. In fact, under our experimental condition, the NB group showed significantly higher glide distance and surfacing distance values compared to the BS group, thus proving both a better sliding immediately after the push from the wall and a longer underwater displacement to the resurfacing point.

Mean glide and surfacing distances were shorter than those found by Blanksby et al. (1996) in prepubertal swimmers. These differences can be attributed partially to the different anthropometric characteristics of the subjects recruited in each study, to measurement techniques and to skill level of the swimmers, from which the conscious decision of choosing the point at which to resume stroking depends (Blanksby et al., 1996).

Previous literature supported the importance of the underwater phase demonstrating how the lengthening of this phase is crucial in reducing total turn time (Blanksby et al., 1996, 1998; Cossor and BR, 2001). Underwater distance has also been shown to be affected by the athlete's ability to maintain a streamlined position during the underwater phase, proving inexperienced swimmers less proficient at streamlining than elite ones (Blanksby et al., 1996; Nicol et al., 2019). Our data indicate that an increased speed off the wall enabled NB to hold the glide further and to resume swimming later than BS. However, the speed-out between the two groups was not statistically different. It has been shown that a significant negative correlation exists between the surfacing distance and the swim resumption speed, i.e., the speed-off (Blanksby et al., 1996). Swimmers who glide too long after push-off will decelerate to less than their average swimming speed. On the whole, this observation suggests that prepubertal swimmers might have less experience in feeling the best point at which to resume swimming after the turn, thus failing to maximise the propulsive force from the wall, losing some of the push-off benefits.

### Effects on Total Turn Time

As a result of all these significant changes shown in kinematic parameters of the different phases of the turn, total turn execution time in the NB group was reduced. Successful performance in short-course races has been shown to depend on the effectiveness of the turn execution time (Slawson et al., 2010; Webster et al., 2011; Chakravorti et al., 2012). In the present work, the total turn time over 5 m was chosen as a benchmark for analysing turn performance, as all fundamental aspects of the turn technique are incorporated within this distance (Blanksby et al., 1996). Our results showed that the decrease in execution times and the higher speeds during the approach and tumble phases, together with the longer underwater displacement following the non-breathing condition, reduced the total turn time.

It is noteworthy that, as the subjects recruited in this study had similar swimming skills and experience, the improvement in turn execution time can be attributed to the specific breathing feature adopted during the approach phase.

### Correlation Analysis

The correlation analysis showed a negative relationship between total turn time and either speed-in or swim-in distance. This suggests that a faster approach to the wall and a rotation of the body farther away from the wall, reduced turn execution time. At the same time, the significant positive relationship between swim-in time and rotation time, and the negative relationship between swim-in time and glide distance suggest that a shorter time of approach to the wall allows a quicker rotation and a longer slide during the underwater phase. Finally, surfacing distance showed a negative correlation with total turn time. This observation is in agreement with a recent study that showed that longer underwater distances were associated with faster turns, confirming the importance of this variable as one of the best predictors of turn performance (Nicol et al., 2019).

In conclusion, in prepubertal swimmers not breathing at the last stroke during the approach phase positively affected the kinematic parameters of the turn, allowing a faster approach to the wall, a quicker rotation of the body, an increased push-off speed, and a shorter turn time, thus improving overall turn performance.

Nevertheless, some limitations are worth noting. First of all, future studies could adopt a crossover design, to confirm results while changing experimental conditions for each participant. Moreover, each subject performed experimental tests on the same day. Future works should repeat tests for each subject on different days, to rule out the possibility that day-to-day variation in physical fitness and performance influences the results. Moreover, further studies are needed to investigate what race distances can benefit the most from this breathing condition during the freestyle turn.

The results of the present study offer useful information and important practical applications for coaches in order to analyse turn kinematic parameters that most characterized turn performance in prepubertal swimmers. In particular, coaches should take into account that not breathing at the last stroke during the approach phase before turning allows their prepubertal swimmers to reduce the turn execution time. This aspect could be particularly relevant in short-course races (i.e., 50–100 m), where turn technique is crucial for the success of the competition. Another important practical application deriving from this study is the low-cost equipment used in the experimental design, easily applicable to all swimming pool contexts. It would be advisable, in the future, to encourage the implement of video analysis as a monitoring tool during training to give coaches detailed information on their swimmers' skill level.

## Data Availability Statement

The raw data supporting the conclusions of this article will be made available by the authors, without undue reservation.

## Ethics Statement

The studies involving human participants were reviewed and approved by Comitato Etico per la Ricerca di Ateneo, Genoa, Italy, No. 2020/21. Written informed consent to participate in this study was provided by the participants' legal guardian/next of kin.

## Author Contributions

EF and MB contributed conception and design of the study and critically discussed the results. LS, SO, and LP performed the experimental study. AB, MP, and CL organized the database and performed the data analysis. EF and AB wrote the first draft of the manuscript. VF and PR wrote sections of the manuscript. All authors contributed to manuscript revision, read, and approved the submitted version.

## Funding

This work was supported in part by the University of Genoa (FRA 2019).

## Conflict of Interest

The authors declare that the research was conducted in the absence of any commercial or financial relationships that could be construed as a potential conflict of interest.

## Publisher's Note

All claims expressed in this article are solely those of the authors and do not necessarily represent those of their affiliated organizations, or those of the publisher, the editors and the reviewers. Any product that may be evaluated in this article, or claim that may be made by its manufacturer, is not guaranteed or endorsed by the publisher.
